# Emotions Represent Evaluative Properties Unconsciously

**DOI:** 10.1007/s10670-024-00873-w

**Published:** 2024-10-16

**Authors:** Constant Bonard

**Affiliations:** University of Bern, Bern, Switzerland

## Abstract

Drawing on affective sciences, I argue that normally elicited emotions involve a component—the appraisal process—that represents evaluative properties unconsciously. More specifically, I argue that, given a substantial agreement in affective sciences about what emotions are, given broadly shared definitions of representation, evaluative properties, and unconsciousness, given how appraisals are conceptualized by most (neuro)psychological theories of emotion, and given empirical evidence about affective states elicited by stimuli perceived unconsciously, we are led to conclude that normally elicited emotions involve a component that represents evaluative properties unconsciously. In the last section, I assess which philosophical theories of emotions are in contradiction with my conclusion, ask whether it implies that emotions represent evaluative properties consciously as well, and discuss how it relates to the debate on unconscious emotions.

## Introduction

Whether emotions represent evaluative properties or not is a polarizing issue in contemporary philosophy of emotion (see 4.1 below). Typically, however, it is not discussed whether emotions represent evaluative properties consciously, unconsciously, neither, or both. Philosophers usually concentrate on conscious representations, sometimes implicitly, and the alternatives are left unexplored. By filling this gap, this article purports to contribute to the literature on the intentionality of emotions and how it relates to evaluative properties.

I argue that normally elicited emotions represent evaluative properties unconsciously. I do so by gathering evidence from diverse kinds of literature that are usually not considered together. More specifically, I argue that, given a substantial agreement in affective sciences about what emotions are (2.1), given broadly used characterizations of representation (2.2), evaluative properties (2.3), and unconsciousness (2.4), given how appraisals are conceptualized by most (neuro)psychological theories of emotion (3.1), and given empirical evidence about affective states elicited by stimuli perceived unconsciously (3.2), we are led to conclude that normally elicited emotions involve a component—the appraisal process—that represents evaluative properties unconsciously (3.3–3.5). In the final section (4), I assess which contemporary philosophical theories of emotions from the aforementioned debate are in contradiction with the conclusion of my argument (4.1). I also ask whether this conclusion implies that emotions represent evaluative properties consciously as well—we will see that this conditional question is a promising thread to follow but deserves further analysis (4.2).

Let me note at the outset that the issue discussed in this paper is independent of, and does not presuppose a stance on, whether emotions themselves can be unconscious (see e.g. Prinz, 2005; Arnaud, 2023). This is because I focus on the unconsciousness of an emotional component—the appraisal process—and not on the unconsciousness of emotions as a whole. Thus, for instance, it is entirely possible to maintain that emotions cannot be unconscious and that appraisals can, as e.g. Clore (1994) does. In 4.3, I elaborate on these remarks and discuss ways in which the argument and conclusion of this paper relate to the debate on unconscious emotions.

## Emotion, Representation, Evaluative Properties, and Unconsciousness

In this part, I characterize the relevant terms.

### Emotion

Despite the absence of a consensus on how to define ‘emotions’, there is substantial agreement within the affective sciences concerning the *components* that emotions are paradigmatically made of across different theories. In the *Annual Review of Psychology*, Scherer and Moors write:

‘There is … substantial agreement [among emotion theorists] that emotional episodes comprise different components such as [1] appraisal of the situation, [2] action preparation, [3] physiological responses, [4] expressive behavior, and [5] subjective feelings.’ (Scherer & Moors, 2019, p. 721)

I will follow this agreement and call ‘emotions’ the physio-psychological episodes that paradigmatically comprise these five components. Let me say a few words about each.

1. *Appraisal of the situation*. This is the process of evaluating how stimuli relate to our goals, understood broadly to include needs, concerns, values, desires, etc. The outputs of this process are called ‘appraisals’.
2. *Action tendency* (or *action preparation*). Emotions typically involve a modification in one’s action tendencies: being ready to flee, fight, give in, etc.
3. *Physiological responses*. Action tendency is inherently linked with physiological changes such as modifications in heart rate, blood pressure, skin conductance, digestion, pupil dilatation, etc.
4. *Expressive behavior*. These are bodily changes that can be controlled to an extent (unlike physiological changes) and that display emotions through one’s face, voice, posture, etc.
5. *Subjective feelings (or phenomenal character)*. This is the experience typical of undergoing an emotion. What it is like to undergo fear or joy.

Note that the agreement is not that any one of these components is *essential* to emotions. There could be non-paradigmatic emotional episodes that don’t involve one or perhaps several of these components. For instance, it is generally agreed that there can be abnormally elicited emotions—e.g. emotions elicited artificially through chemical or electrical stimulations—that don’t involve appraisals (for a review, see Moors, 2022, pp. 262–264).

Because it will be central to my argument, let me say more about appraisals already. Appraisals have been posited to explain a straightforward phenomenon: the absence of one-to-one relations between kinds of stimuli and kinds of emotional responses. The same kind of stimuli—e.g. a mouse running in the kitchen—can elicit different kinds of emotions—e.g. fear vs anger—and different kinds of stimuli—e.g. seeing a snake vs seeing a red rose—may elicit the same kind of emotion—e.g. aesthetic admiration. This many-to-many relation cannot be explained by the simple, automatic, ‘mechanical’ stimulus–response analysis that we can give for other biological reactions, such as reflexes or white blood cells’ behavior. In the latter cases, we find one-to-one relations between kinds of stimuli and kinds of responses. For instance, if a neutrophil white blood cell enters into contact with certain chemicals (stimulus), it will follow its source to approach the site of inflammation (response). Similarly, if you hit a functioning leg at a certain place with a certain force (stimulus), you’ll trigger a reflex reaction (response).

The absence of such one-to-one relations in emotions led researchers to posit appraisals as an intermediary psychological process. For instance, someone reacts with fear to a mouse in the kitchen because it is appraised as a threat. Someone else reacts with anger because it is appraised as a nuisance to be fought. And someone reacts with aesthetic admiration toward either a snake or a rose because the two kinds of stimuli are appraised in the same kind of way—as fascinatingly beautiful, perhaps.

### Representation

Two kinds of definitions of representation stand out in the contemporary philosophy of mind and cognitive sciences: function-based and accuracy-based definitions. I will allow ‘representation’ to mean either. These are not the only two kinds of definitions, but they should in any case provide a useful reference point to discuss whether emotions represent evaluative properties.

Dretske (1995) gives a broad and flexible function-based definition that is compatible with most, if not all, function-based accounts. Here it is:

‘… a system S represents a property F if and only if S has the function of indicating (providing information about) the F of a certain domain of objects.’ (Dretske, 1995, p. 2)

To illustrate: chromatic perception represents surface reflectance if, and only if, the function of chromatic perception is to provide information about the surface reflectance of a certain domain of objects.

One can accept this definition without being committed to Dretske’s general framework, e.g. to his definitions of function and indication. As such, this definition of representation is compatible, among others, with several teleosemantic accounts, inferential role semantics, and asymmetric dependence theory.

However, some philosophers consider that function-based definitions such as Dretske’s are too broad. Burge (2010, Chapter 9, 2014), in particular, has argued that they inappropriately count states of certain lower-level organisms such as plants and bacteria as representational.

Instead, Burge proposes an accuracy-based account according to which what is distinctive of representations is that they have underived accuracy conditions that play a non-trivial explanatory role in scientific explanations (2010, p. 12, 2014, p. 393).

He gives visual perception as an example where representational states have been postulated by scientists to explain the underdetermination problem, i.e. the problem of explaining how perception manages to, usually correctly, disambiguate between stimulations of perceptual organs that could be identical in different situations. For instance, when we move toward an object, the way the light hits our retina may be identical to a situation where the object’s shape gets bigger. Perceptual psychology must explain how ambiguous proximal stimulations cause unambiguous, usually correct, perception. It does so by positing representational states (Burge, 2014, p. 398). Perceiving the size of an object as constant despite the changes in retina stimulations and other such perceptual constancies are explained by the stability of representations of external objects, representations that are formed by eliminating the ambiguity of proximal stimulations (Burge, 2010, pp. 397–398). Such representations possess accuracy conditions in the sense that only some possible ways the world could be are in accordance with them. When we move toward an object, the perceptual representation is accurate only if our vision represents the size of objects as constant despite changes in proximal stimulations. These accuracy conditions are not derived from those of other states: the representations in question are their primary source. In sum, such states possess underived accuracy conditions which play a non-trivial explanatory role in scientific explanations. This is what makes them representations, according to Burge.

In 3.3, I will argue that appraisals are representations according to both these function-based and accuracy-based definitions.

### Evaluative Properties

By ‘O possesses an evaluative property’ I mean that O is—relative or not to an individual or to a kind, finally or not (for reviews of these distinctions, see Schroeder, 2016; Zimmerman, 2015)—positive or negative morally, hedonically, aesthetically, epistemically, cognitively, politically, instrumentally, socially, prudentially, or in another axiological way. In short, O is positive or negative in some value-laden way.

Note that evaluative properties may be characterized more precisely, for instance in terms of moral duties, norms, reasons, or what is right (see e.g. the review in Zimmerman, 2015, Sect. 1.2). I refrain from doing so because I want to leave the relation between evaluative properties and normativity open, e.g. whether all instances of evaluative property imply that some agent thereby has some duty, has a normative reason to have an attitude, is subjected to a norm to act, etc. My characterization also remains neutral concerning the ontological nature of evaluative properties and whether they should be accounted for by subjectivism, naïve realism, cultural relativism, error-theory, fitting-attitude analysis, or another theory.

Let me note already that my argument leads to the claim that what kinds of evaluative properties are unconsciously represented by emotions is an empirical issue. However, my argument remains entirely neutral concerning what kinds of evaluative properties are the correct objects of emotion kinds, which is a normative issue—‘formal objects’ is sometimes used for evaluative properties under both these descriptions, which can be confusing. I will discuss in 3.4 whether what I take to be the evaluative properties unconsciously represented by emotions correspond to those usually discussed by philosophers of emotion.

### (Un)consciousness

Consciousness is sometimes equated with the phenomenal character of mental states, i.e. the what-it-is-like or qualitative aspect of consciously seeing, hearing, imagining, fearing something, and so on. However, some contemporary theories do not equate consciousness with phenomenal character. To characterize what is meant by ‘consciousness’ below, let me illustrate this issue by briefly describing four influential theories.

Attended Intermediate Representation theory (Prinz, 2012) posits that we consciously experience only ‘intermediate representations’, which concern basic features of objects, such as colors, shapes, tones, or feels. But it is only when these intermediate representations are attended that they become conscious, are felt, and acquire their phenomenal properties. Thus, for this theory, there cannot be unconscious states with phenomenal characters or conscious states without phenomenal characters.

According to Global Workspace theories, becoming conscious of some information from one’s mental states happens when that information is widely shared across the brain (Baars, 2017; Dehaene et al., 2011). In Dehaene’s version, this global activity creates our subjective experience so that phenomenal character is inseparable from consciousness. Furthermore, the possibility of unconscious mental states with phenomenal characters is considered an untestable hypothesis (Naccache & Dehaene, 2007).

By contrast, Higher Order theories posit that a mental state (perception, memory, imagination, reasoning, …) becomes conscious only when we are thinking about it (Rosenthal, 1991), perceiving it (Armstrong, 1981), or engaging with it with some other mental state (Carruthers, 2004). In such theories, notably in Rosenthal’s version, there is a possible separation between phenomenal character and consciousness since there can be, in principle, mental states with phenomenal characters that are not the object of the relevant higher-order thought (Rosenthal, 1991).

Recurrent Processing theories (e.g. Block, 2002) also distinguish phenomenal character, which it calls ‘phenomenal consciousness’, from what it calls ‘access consciousness’. Here is Block’s characterization of being access-conscious or ‘A-conscious’: ‘A representation is A-conscious if it is broadcast for free use in reasoning and for direct ‘rational’ control of action (including reporting).’ (Block, 2002, p. 208) Here is an example to which I will come back below. Blindsight patients possess a blind spot in their visual field and cannot consciously perceive objects situated in this hole. They are not A-conscious of these objects because their representation is not freely, directly available to them for reasoning and action. However, surprisingly, if they are forced to guess what is in their blind field, they will guess correctly above chance level. They will think that they are randomly guessing, but the above chance level shows that they form A-unconscious representations of these objects. According to Recurrent Processing (or Higher Order) theories these A-unconscious representations nevertheless possess the phenomenal character of vision.

So, according to the last two theories reviewed, unlike Prinz’s and Dehaene’s theories, there can be mental states that have a phenomenal character but that are not conscious, in some sense of ‘conscious’. I will not presuppose a stance on this question. I want to stay as neutral as possible concerning these and other contemporary theories of consciousness. Thus, in the following, I will use ‘unconscious representations’ to refer to representations that are not accessible consciously and stay neutral on whether they may or not possess a phenomenal character.

## How Emotions Unconsciously Represent Evaluative Properties

In this part of the paper, I argue that normally elicited emotions represent evaluative properties unconsciously.

### Appraisals as Unconscious: Theoretical Evidence

I start with a reason for thinking that appraisals are unconscious. I call it the ‘theoretical evidence’. Here it is: the appraisals that contemporary (neuro)psychological theories posit as components of all normally elicited emotional episodes don’t correspond to the mental processes to which we have direct conscious access during the elicitation phase of emotional episodes.

In the early twentieth century, emotion theorists postulating appraisals saw them as regular evaluative judgments that are accessible directly through conscious experience and introspection (Reisenzein, 2006). It has later been argued that appraisals can be realized in several different ways (see e.g. Leventhal & Scherer, 1987). Researchers today disagree about whether and which appraisals are hardwired, culturally variable, sequentially processed, language-dependent, and other ways in which appraisals can be realized. As far as the present argument is concerned, the relevant dimension of variation is between appraisals that are realized consciously or unconsciously (though I also discuss hardwiredness in 3.3).

Now, it is usual in the contemporary literature to distinguish between two broad cognitive levels of appraisals: the basic appraisals being unconscious (and are also usually considered as fast, automatic, error-prone, and associated with the limbic system, especially the amygdala) and the higher-level appraisals being conscious (and slower, more flexible, more accurate, and associated with prefrontal cortical activities) (Frijda, 2007; Kalisch et al., 2006; Leventhal & Scherer, 1987; Sander et al., 2018). Neuroimaging data suggest that the basic appraisals are always present in normal emotional episodes while the higher-level ones are not (Kalisch et al., 2006). The considerations in this section also constitute an indication that whenever higher-level appraisals are instantiated, basic-level appraisals are too. The component to which I referred above (2.1) that is postulated to be present in all paradigmatic emotion episodes by most affective scientists is the basic (fast, automatic, error-prone, limbic) appraisal process. ‘Appraisals’ without qualification will thus refer to basic appraisals.

Reviewing appraisal theories, Sander et al. (2018) highlight the wide agreement that the appraisal process involves ‘answers’ to all of the following questions (I use square quotes because these ‘answers’ may well have a nonlinguistic format):

‘How relevant is this event for me?; Does it directly affect my social reference group or me? (goal relevance); What are the implications or consequences of this event and how do these affect my well-being and my immediate or long-term goals? (goal congruence); Did I expect this event and its consequences and how certain are they (novelty, expectation, certainty); Who caused this event, am I responsible or someone else? (agency, causation); How well can I cope with or adjust to these consequences? (coping potential, control, power).’ (Sander et al., 2018, p. 226)

Since the appraisal process explains the elicitation of emotional episodes, these questions are to be answered in the blink of an eye: less than 250 ms after the onset of the stimulus with the first answers happening in less than 50 ms (Grandjean & Scherer, 2008). But the flow of our consciousness during the first tenths of a second of an emotional episode does not consist in, nor does it include, the conscious impression that we answer these questions. Instead, these appraisals are supposed to correspond to categorizations that we would need to postulate to predict reactions and behaviors: they are not supposed to correspond to consciously accessible mental states—as such, they are comparable to the unconscious representations postulated to explain blindsight experiments (2.4).

The appraisal theories reviewed by Sander et al. (2018), although well-established and influential, are not the only (neuro)psychological theories of emotion. Aren’t there influential theories in this field that don’t conceive of basic appraisals as unconscious by default? Not that I know. Besides the appraisal theory, the main (neuro)psychological theories of emotion are the psychological construction, the basic emotion, and the somatic marker theories. These three theories also hypothesize or imply that basic appraisals are unconscious by default. The major psychological construction theories are committed to the existence of an appraisal process as it is understood here since they hold that specific emotions can be distinguished from each other based on the psychological situations in which they arise (Ortony & Clore, 2015, p. 314). The appraisal process is antecedent to ‘core affects’, which is the first element that is consciously perceived during an emotional episode for constructivists. Core affects are themselves antecedent to prototypical emotional episodes as the latter require a further interpretation of core affects (‘labelization’) (Barrett, 2006; Ortony & Clore, 2015; Russell, 2003). Therefore, the appraisal process is antecedent to what is conscious in emotions in this theory too—though it may not use the term ‘appraisal’. Similarly, current basic emotion theorists consider the appraisal process as ‘immediate, unbidden, opaque, *unconscious*, and automatic’ (Matsumoto & Ekman, 2009, p. 107, my italics). Finally, the somatic marker hypothesis also implies that the feeling component of emotions results from the appraisal process as I defined it: antecedent states that include the brain processes explaining which kind of emotion will result from the detection of a given stimulus (Damasio, 1998).

In sum, the dominant current (neuro)psychological theories lead to consider that, by default, the appraisal process is unconscious. That is, there is an unconscious appraisal process in all normally elicited emotional episodes though this process may sometimes partially emerge in consciousness (e.g. Grandjean et al., 2008). I discuss this ‘emergence’ to consciousness below.

### Appraisals as Unconscious: Experimental Evidence

The theoretical evidence discussed so far is supported by results from several experiments that I will now discuss. Though this experimental evidence is not by itself sufficient to argue that emotional episodes in general involve unconscious appraisals, the data discussed in this and the preceding sections mutually reinforce each other.

In an influential study, Öhman and Soares (1994) elicited negative affects through subliminal pictures. Pictures of snakes and spiders as well as of flowers and mushrooms were projected for a time too brief to be consciously accessible (20 and 30 ms). Subjects could not guess above chance which pictures they saw when asked in a forced-choice task. Nevertheless, subjects who unconsciously saw snakes or spiders reported feeling, overall, significantly more negative, more aroused, and less dominant than those presented with mushrooms or flowers. Such feelings are paradigmatically associated with fear. This indicates that subjects appraised snakes and spiders as negative stimuli unconsciously.

Other experiments show that subliminal exposure to pictures influences action tendencies. In one of them (Winkielman & Berridge, 2004), participants were subliminally exposed to either happy or angry facial expressions and were then asked to taste a beverage and evaluate it. The ones exposed to happy faces drank more and were willing to pay twice as much for the drink. This indicates that participants were unconsciously appraising the situation positively or negatively depending on whether they were subliminally exposed to happy or angry faces.

One can easily multiply examples of experiments showing how stimuli that are not consciously accessible can influence affective states, and modify action tendencies, physiological changes, and neuronal processes associated with emotions (see notably the review in Smith & Lane, 2016). Because the elicitation of these affective episodes is best explained by postulating an appraisal process, these experiments indicate the presence of unconscious appraisals.

One could respond that, in all these experiments, since there are no conscious intentional objects, there may be no intentional objects at all, but since emotions are always intentional, the affective states in question are not emotions. They may be moods, perhaps (as I discuss in Bonard, 2022). If that is so, how could these experiments tell us anything about the appraisal process involved in *emotions* as opposed to moods or other kinds of affective states?

It is highly dubious that the participants in these experiments are in non-intentional states. They respond to the unconscious stimuli in a way that is identical or very similar to how they respond when they consciously perceive the same stimuli. The straightforward explanation is that the unconscious stimuli (e.g. the subliminal pictures) constitute unconscious intentional objects.

Furthermore, even if we were to grant that these experiments don’t feature emotional episodes but rather another kind of affective episodes such as moods, they nevertheless constitute evidence that the appraisal process involved in emotions can be unconscious insofar as it is more than reasonable to hold that the relevant mental mechanisms involved in the affective episodes in question are the appraisals involved in emotions. Indeed, since evolution usually is parsimonious, it would be strange that we possess two cognitive mechanisms whose roles are the same: detecting and reacting to goal-relevant situations (e.g. threats such as snakes, spiders, and angry people) by modifications of physiological changes, action tendencies, and/or of affective feelings (e.g. negative valence, high arousal, feeling powerless). It is theoretically possible, but not plausible, that there are two distinct mental mechanisms with identical functions that respond to the same stimuli with the same outputs. The hypothesis that the relevant mental mechanisms involved in these experiments are the appraisal processes that we have discussed in the last sections is much more parsimonious, especially because we saw that current psychological theories hypothesize that basic appraisals are not conscious.

At this point, one could ask: Don’t the experiments discussed show that, in some cases, we have evidence for the basic, fast, unconscious appraisals but that, in other cases, we could have higher-level, conscious appraisals without the basic, unconscious ones? Take, for instance, someone who is joyful because they have solved a difficult mathematical problem. They may think to themselves ‘It is awesome I solved this problem’. This is a conscious, higher-level appraisal. How would we know whether such higher-level appraisals are always accompanied by lower-level ones? Why shouldn't  we think that, in such cases, it is the higher-level, conscious appraisals that trigger the emotions instead of lower-level, unconscious ones?

A main reason to think so comes from what I called the ‘theoretical evidence’ above. We don’t have a conscious access to the appraisal process as contemporary theories conceptualize it. Let me elaborate.^1^

Remember the ‘many-to-many’ rationale for appraisals discussed in 2.1. Appraisals were postulated in large part to explain how different types of stimuli trigger the same type of emotional reaction and how the same type of stimuli trigger different types of emotion. To each emotion type—joy, anger, sadness, etc.—corresponds an appraisal profile. Thus, the joy in solving a complex mathematical problem is triggered by an appraisal process consisting of the same ‘answers’ to the same ‘questions’ as in all other cases of joy. No matter if the object of the emotion is solving a difficult mathematical problem or satisfying one’s hunger, as long as it is the same type of emotion, the appraisals are supposed to be of the same type.^2^

Now, in the mathematical problem example, the joyful person does not have the impression to ‘answer’ anything like the questions listed in the Sander et al., (2018, p. 226) quote from the preceding section. The person thinks to themselves ‘It is awesome I solved this problem’ and not ‘This event is highly relevant for me; It directly affects me; The implications of this event for my well-being and my immediate or long-term goals are positive; I did not expect this event; I am responsible for causing this event; I can cope with the consequences well’.

If asked, the person may agree that the appraisals in question correspond to how they see the event, but the flow of their consciousness in the milliseconds separating their realization that the problem was solved and the emergence of their feeling of joy certainly did not involve a conscious access to these appraisals. It is not like, before our emotions are triggered, we have the impression of going through such a list of appraisals. However, according to contemporary appraisal theories, our mind does go through such a list. Thus, we don’t have conscious access to the appraisals postulated by contemporary appraisal theories.

If one nevertheless wants to hold onto the idea that it is not an unconscious appraisal process that triggers the joy but rather the conscious thought ‘It is awesome I solved this problem’, I see two options. First, one can reject the ‘theoretical evidence’ and, in particular, the idea that emotions result from ‘answers’ to the set of questions listed above. Alternatively, one can explain how conscious thought such as ‘It is awesome I solved this problem’ *consists in* the appraisal process for joy postulated by contemporary theories.^3^ I don’t see, however, how one could defend either one of these two options. I thus consider them as less plausible than the one I presented, namely that solving the mathematical problem triggers an unconscious appraisal process that, in turn, triggers a feeling of joy accompanied by conscious thoughts which partially reflect the unconscious appraisal process.

Moreover, neuroimaging data also provides support for the idea that the basic, low-level, appraisal process that I described is unconscious by default although it may partially emerge into consciousness in some cases in the form of higher-level appraisals such as the thought ‘It is awesome I solved this problem’. As mentioned above, higher-level appraisals are associated with prefrontal cortical activities (among others) while lower-level appraisals are associated with limbic activities, especially with the amygdala—the latter being associated with appraisals of goal-relevance in all kinds of emotions. Now, neuroimaging studies indicate that higher-level/cortical appraisals are always accompanied by lower-level/limbic appraisals (Kalisch et al., 2006; Murray et al., 2022).^4^ Of course, there presumably always are unconscious processes that subtend conscious ones—this is rather trivial. My point, however, is much more specific: I argue that the unconscious process in question—the low-level, unconscious appraisal process posited by contemporary theories—represents evaluative properties. This is far from trivial insofar as it is not obvious and, if true, it bears on several philosophical issues on emotions (see 4).

### Appraisals as representations

Now that we know more about appraisals, it should become clear that, according to the function-based account of representations proposed notably by Dretske and discussed above (2.2), appraisals very plausibly are representations: states of a system that have the function of indicating that something is the case. Very plausibly, appraisals have the function of indicating that certain stimuli possess goal-relevant properties because this helps us react to these stimuli in useful ways, *ceteris paribus*.

To see why, assume along with many emotion theorists that, when one undergoes fear about a stimulus, one appraises it as urgently unconducive to one's safety and as hard to control. The function of such appraisals is rather clear: if the stimulus is indeed urgently unconducive to one’s safety and hard to control, one would better do something about it; one should react to this stimulus accordingly. This categorization is especially advantageous because there is a type of reaction that is generally useful toward all kinds of stimuli that are urgently unconducive to one’s safety and hard to control: avoid them. Fear allows such a reaction in a very efficient way—our blood flow circulates more quickly so that we may deploy our muscles more efficiently; our eyes and nostrils open up to gather more information; adrenaline rushes make us more alert, focus our attention, and stop our digestive process to save energy; etc. Countless other physiological changes prepare us for an avoidance reaction to the threat. Furthermore, since stimuli that are urgently unconducive to one’s safety and hard to control are involved in a vast variety of different kinds of situations (e.g. being too close to a snake vs. to a cliff vs. to a falling tree), a mechanism whose purpose is to yield a fast, automatic categorization of stimuli as possessing these properties is an enormous advantage for the individual—and, because one better be safe than sorry, that is so even if this mechanism is error-prone and yields many false alarms. The appraisal functions to indicate that the stimuli possess goal-relevant properties because this helps to react in useful ways.

Thus, appraisals are plausibly conceived as possessing the function of indicating that certain states of affairs obtain. More precisely, the function of indicating that certain stimuli possess properties that are relevant to our goals and to which we should react with the action-tendencies of the emotion kind that is associated with the appraisals in question, e.g. (roughly) the avoidance of fear, the hostility of anger, the repulsion of disgust, the celebration of joy, etc. (Deonna & Teroni, 2012, Chapter 7; Frijda, 2007; Scarantino, 2014).

As we saw though (2.1), some argue that an indicative function is not sufficient for representation because it may unwarrantedly ascribe representational capacities to simpler organisms such as plants and bacteria (Burge, 2010, 2014). Now, some of these organisms possess mechanisms that help them avoid stimuli that are urgently unconducive to their safety and hard to control. For instance, certain trees retract their leaves to protect themselves when they detect fire and some bacteria move away from toxic chemicals. Based on such observations, one may claim that emotion elicitation may be explained without representations since the defense mechanism of bacteria and plants is very similar to fear responses but that they can be explained without representations.

However, unlike fear responses, the defense mechanisms of plants and bacteria function through one-to-one correlation: the reactions in question are limited to a given type of stimuli. By contrast, as we saw, the appraisal process involved in emotions applies to vastly different types. Not only can the stimuli triggering fear possess drastically different perceptual properties—e.g. a snake vs. a cliff vs. the sound of an explosion—but they may not be perceptible at all—e.g. a financial crisis. Furthermore, the list of such stimuli is expandable: completely new stimuli may be appraised as urgently unconducive to one’s safety and hard to control—e.g. the COVID-19 virus. Finally, the list depends on one’s goals, which vary across individuals and over time—e.g. Sam’s friends will fear losing him, but Sam’s archenemy may rejoice at his death.

These points indicate that psychological theories, by postulating appraisals, posit a process that corresponds to Burge’s definition of representation (2.2). First, appraisals require stable internal categorizations: a given pattern of appraisals—e.g. the ones associated with fear responses—is constant across different kinds of proximal stimulations. In fact, it must be stable beyond proximal, perceptual stimulations since stimuli that are not perceived can also trigger an emotion—fear, for instance, can be triggered by thought processes such as deductive inferences. Thus, appraisals, to play the relevant explanatory role, must be stable internal categorizations of stimuli across this enormous variety. Now, these categorizations play a non-trivial explanatory role in scientific explanations: they are needed to explain why one reacts with one type of emotion to vastly different types of stimuli and why there can be different kinds of emotional responses to the same kind of stimuli in individuals of the same species. Finally, appraisals possess underived accuracy conditions: only some possible ways the world could be are in accordance with them. For instance, if fear involves appraising the stimuli as urgently unconducive to one’s safety and as hard to control, then only stimuli that possess such properties are in accordance with this appraisal. In short, appraisals possess underived accuracy conditions that play a non-trivial explanatory role in scientific explanations. They thus meet Burge’s requirements for being representations. Anecdotally, but I think tellingly, Burge himself gives as an example of a representational state a rabbit’s (mis)perception of danger (2014, p. 395).^5^

So, at least in the sense of the two popular accounts of representations discussed in 2.2, we can conclude that appraisals are representational states.

Let me briefly address an objection related to this claim: Aren’t there emotional reactions that are linked in a one-to-one relation to kinds of stimuli because evolution has shaped our body or hardwired our brain in this way? Candidates involve fear-like responses to spiders, snakes, heights, and strangers (LoBue & Adolph, 2019). In these cases, there may be no appraisal process going on since there would not be any need for an appraisal process in the absence of many-to-many relations between kinds of stimuli and kinds of emotions. The emotional response would not involve representations: our body would react to the stimuli in a reflex-like way, as with the defense mechanisms of trees and bacteria. In such cases, according to the objection, the emotions would result from bodily changes, triggered by non-cognitive mechanisms, and not from representations. I will call such hypothetical cases ‘representation-free emotions’.

To respond, let me first observe that the evidence for one-to-one relations between kinds of stimuli and kinds of emotional responses is very much debated. Experiments supporting their existence may not be reliable (LoBue & Adolph, 2019). And in the absence of such one-to-one relations, as we saw above (2.2 and 3.3), we are led to posit appraisals, and thus representations.

Second, even if there are such one-to-one relations, they may be explained with an appraisal process and so without postulating representation-free emotions: what is universal in such cases is the stimulus–appraisal relation, for instance, the relation between snake-looking stimulus and the representation that this is urgently unconducive to one’s safety (this is the solution favored by Prinz (2004), using a Dretskean notion of representation).

Third, we are capable of learning not to have emotional reactions to seemingly all kinds of stimuli, e.g. to not react with fear to snakes, spiders, heights, or strangers. Think for instance of a skyscraper window cleaner whose job does not trigger any fear anymore. By contrast, we cannot learn to suppress our reflexes (e.g. knee jerk) nor can bacteria and trees learn to suppress their defense mechanisms, it seems. This contrast is easily explainable if representations can be modified by learning and if putative representation-free emotions are in fact mediated by representations.^6^

I am not suggesting that emotions are not embodied responses. What I am saying is that embodied responses in emotions are triggered by antecedent unconscious appraisals. For a more detailed discussion of why embodied theories of emotion should posit representations, as I argue here, see Hufendiek (2018).

### Appraisals as Representations of Evaluative Properties

We saw that the appraisals postulated by recent psychological theories should be conceptualized as unconscious representations. But are they representations of *evaluative properties*?

As far as I know, all contemporary theories of appraisals postulate goal-(un)conduciveness appraisals (for reviews, see Lange et al., 2020; Sander et al., 2018; Wharton et al. 2021, sect. 4) and so I will concentrate on these. What types of goals need to be posited to explain emotional reactions is a disputed issue—e.g. some insist on evolutionary relevant circumstances (Ekman & Cordaro, 2011), others on social constructions (Russell, 2003). However, it is agreed that the goals in question are restricted to those that are sufficiently important to the individual. And the conduciveness/unconduciveness must also be sufficiently strong. This is because only what is represented as *strongly* conducive/unconducive to *important* goals can trigger normally elicited emotions.

Now, do these appraisals imply representations of evaluative properties? For example: if I saw a tiger and am afraid because I represent my situation as strongly unconducive to my safety, do I thereby represent it as possessing a negative evaluative property? Or: if I admire your action because I represent it as strongly conducive to fairness—one of my primary concerns—does my appraisal represent your action as possessing a positive evaluative property?

Remember that by ‘O possesses an evaluative property’ I mean that O is positive or negative in some value-laden way, e.g. morally, hedonically, aesthetically, epistemically, cognitively, politically, instrumentally, socially, prudentially, or in another axiological way, relative or not to an individual or a kind, finally or not.

In this sense, it does seem that the representations of, for instance, my encounter with a tiger as unconducive to my safety includes a representation of an evaluative property, since the representation of being strongly unconducive to one’s safety, to an individual that importantly values their safety, appears to be a representation of something as negative prudentially relative to this individual (for similar reasoning, see Tappolet, 2016, pp. 51–52). By that, I am not claiming that the individual possesses the concepts ‘unconducive’, ‘safety’, ‘negative’, and ‘prudential’ and that they make inferences between them. Rather, I mean that the appraisal in question belongs to the set whose members are representations of something as negative in a value-laden way. It does because it belongs to one of its subsets: that whose members are representations of something as negative prudentially relative to an individual.

Well-being has been hypothesized as the goal that is common to all appraisals of goal-un/conduciveness that trigger emotions (Sander et al., 2018, p. 225). So, the relevant evaluative properties can be expected to usually be positive or negative prudentially. However, it is unclear that well-being has a monopoly on the relevant goals. For instance, insofar as you highly value fairness, your representation of something as strongly unconducive to fairness will, I take it, be a representation of something as negative in a value-laden way—even if it is not represented as unconducive to your (or someone else’s) well-being. *Mutatis mutandis* for other goals—and remember that ‘goals’ is used broadly to include concerns, desires, needs, ideals, personal values, etc.

The following question then arises: are the evaluative properties represented by appraisals the same as those in which philosophers have been interested? Philosophical theories of emotion, beginning with Aristotle’s, have generally discussed properties such as slight (for anger), loss (for sadness), injustice (for indignation), and danger (for fear). And I have not explicitly mentioned such properties—besides mentioning ‘danger’ when discussing Burge.

Nevertheless, all that I have said so far is, in principle, compatible with the idea that appraisals represent slight, loss, injustice, or danger. For instance, a representation of something as strongly unconducive to one’s safety may be coreferential with a representation of it as unsafe—and though philosophers may rather use the words ‘danger’ or ‘threat’, it is unclear whether ‘unsafe’ is really different from what they have in mind. Similarly, a representation of X as unfair—which is an evaluative property typically discussed by philosophers—may well be coreferential with a representation of X as unconducive to fairness (perhaps X is unfair iff X is unconducive to fairness). Thus, at least some of the represented properties discussed in psychology may correspond to those discussed in philosophy (on different possible relations that appraisals may have with the kinds of values traditionally discussed by philosophers of emotion, see Teroni, 2021).

Finally, note that we should not exclude the possibility that, in light of empirical findings on appraisals, philosophers would need to revise their hypotheses about the kinds of evaluative properties that the objects of emotions are apprehended or acknowledged as having. Let me highlight however, as mentioned above, that this is different from the claim that philosophers may need to revise, in light of empirical findings, what they take as the *correct* objects of emotions—the latter is a normative matter distinct from the descriptive issue in question.

### Conclusion of the Argument

We have now gathered all the ingredients. Given the substantial agreement in affective sciences about what emotions are (2.1), given broadly shared conceptions of representation (2.2), evaluative properties (2.3), and (un)consciousness (2.4), given how appraisals are conceptualized among current (neuro)psychological theories of emotion (3.1) and empirical evidence (3.2), we are led to conclude that normally elicited emotions probably involve proper parts that represent evaluative properties unconsciously. In short, emotions represent evaluative properties unconsciously.

### But are Appraisals Emotional Components?

I will now address an objection to the premise with which I have begun (2.1): that appraisals are emotional components. The objection is that, contrary to the substantial agreement in affective sciences,^7^ the appraisal process is, at best, an external antecedent cause of emotional episodes, not an emotional component (this objection focuses on normally elicited emotions). I will discuss two reasons to make this objection.

The first reason results from the conjunction of two claims:

- the appraisal process is not specific to emotions
- only what is specific to emotions can be an emotional component

Claim (a) is held by psychological constructivists (e.g. Russell, 2003), belief-desire theorists (e.g. Bonard, under review; Reisenzein, 2009), and goal-directed theorists (e.g. Moors, 2022, Chapter 7). These researchers suggest that what I have called ‘appraisals’ are in fact ordinary cognitive/conative states that are not emotion-specific. By contrast, other theorists hold that appraisals are *sui generis* kinds of representations that are not reducible to general cognitive/conative states (e.g. Matsumoto & Ekman, 2009; Panksepp, 1998, Chapter 3; Sander et al., 2018). As far as I can tell, there is no strong evidence that favors one of these two possibilities. So, claim (a) may be true.

However, (b) is highly dubious: it is not because a cognitive process is not domain-specific that it should not count as a component of this domain. Compare: some of the cognitive processes underlying musical listening are not music-specific because they are also used in verbal listening (Bonard, 2018; Patel, 2010). But this does not mean that they are not cognitive components of musical listening. Similarly, many affective scientists consider that physiological changes, action tendencies, motor behaviors, and indeed cognitive/conative processes are not emotion specific but nevertheless are emotional components.

The conjunction of (a) and (b) is also objectionable insofar as it commits to the idea that emotions are nothing but feelings. Indeed, if (a) is correct, (b) implies excluding not only appraisals from emotional episodes, but all components that are not emotion-specific. Since there seem to be no action tendencies, behaviors, or physiological changes that are specific to emotions, and the only component that is specific to them is a subpart of the feeling component, (a) and (b) leads to the claim that emotions are only constituted by it. However, we should allow for the possibility that the widespread consensus introduced in 2.1 is at least partly correct and that emotional episodes can be constituted by other components: plausibly, emotions involve bodily changes, they involve action tendencies, and they involve motor expressions. So (a) and (b) should not lead us to deny the inclusion of appraisals.

Let me turn to the second, and more fundamental, reason for denying that appraisals are emotional components. The substantial agreement that appraisals are emotional components may be due to a particular metaphysical view. For instance, it is plausible that a form of psycho-functionalist metaphysics is presupposed by many affective scientists. Thus, appraisals may be considered emotional components *because* they are hypothesized to play a psychological function attributed to emotional processes. But, even if one recognizes the existence of appraisals as I have construed them, metaphysical psycho-functionalism is not the only game in town. For instance, a type of metaphysical phenomenalism may hold that we should identify emotions solely based on their particular phenomenal characters and so insist that unconscious appraisals are not proper emotional components (though unconscious appraisals may nevertheless have particular phenomenal characters, see 2.4). And certain enactivists may construe emotions as observable, response-based, whole-organism, engagements with one’s environment and thus insist that unconscious appraisals should not be considered as constitutive of what emotions really are, but at best non-mental antecedent events.

In general, one may agree that appraisals are unconscious representations of evaluative properties that accompany all normally elicited emotional episodes but refuse to count them as emotional components because of their metaphysical overarching theory. They would agree about the stuff making up reality but disagree about how to carve it.

The metaphysical question of how to carve mental and nonmental reality and whether some form of metaphysical psycho-functionalism, phenomenalism, enactivism, or another view is correct is a complex and deep issue that, of course, cannot be resolved here. There is no knock-down argument for one of these views. I thus recognize the force of this objection.

However, this matter of fact nevertheless leads to a practical reason to use ‘emotion’ for episodes including appraisals: only strong arguments could convince emotion researchers to change the way they use the word ‘emotion’ and it would be preferable that we all use ‘emotion’ to refer to the same phenomena. If there are no strong enough arguments to reject the wide agreement in affective sciences according to which appraisals are emotional components, this is a practical reason for this use of ‘emotion’. But because the metaphysical debate is indecisive, arguments about how to carve mental reality don’t constitute strong enough arguments.

Of course, this practical reason only has some bite in a context where widely held theories and presuppositions lead most emotion researchers to consider appraisals as emotional components. Future affective sciences may constitute a different context in light of forthcoming findings and insights. However, for now, the burden of proof seems to be with those holding that the appraisal process is not part of the emotion but an external antecedent cause of it.

## Implications

In this concluding section, I turn to some potential implications of the claims made above concerning three philosophical issues.

### Emotion, Representation, and (Un)consciousness

In the introduction, I remarked that the question ‘Do emotions represent evaluative properties?’ is a polarizing issue in contemporary philosophy of emotion. The discussion above can hopefully help to make some progress in this debate by clarifying it. In particular, it reveals that there are pairs of theories that (appear to) contradict each other in answering the unqualified question—i.e. ‘Do emotions represent evaluative properties?’—but which don’t contradict each other once we take a consciousness/unconsciousness distinction into account. For instance, the apparent contradiction on this question between motivationalism and charitable^8^ interpretations of attitudinalism and non-intentionalism may be overcome (though they differ on other questions). The positions in question are represented in Table 1 below.^9^

**Table 1:** Do emotions represent evaluative properties consciously, unconsciously, both, or neither?

Do emotions represent evaluative properties…	Yes	Unspecified	No
…Without qualification?	(Quasi-)judgmentalism (Nussbaum, 2001; Roberts, 2003; Solomon, 1993); (Quasi-)perceptualism (de Sousa, 1987; Döring, 2007; Goldie, 2002; Helm, 2009; Prinz, 2004; Ratcliffe, 2005; Roberts, 2003; Tappolet, 2016); Motivationalism (Scarantino, 2014); Representationalism about affective experiences (Mendelovici, 2014; Tye, 2008); Representationalist enactivism (Hufendiek, 2018)		Attitudinalism (Deonna & Teroni, 2012); Non-intentionalism (Shargel, 2015; Whiting, 2011); Non-representational enactivism (Hutto, 2012; Shargel & Prinz, 2018)
…Unconsciously?	Motivationalism (Scarantino, 2014); Prinz’s (2004) perceptualism,	(Quasi-)judgmentalism (Nussbaum, 2001; Roberts, 2003; Solomon, 1993); (Quasi-) perceptualism except Prinz’s (2004) (de Sousa, 1987; Döring, 2007; Goldie, 2002; Helm, 2009; Ratcliffe, 2005; Roberts, 2003; Tappolet, 2016); Attitudinalism (Deonna & Teroni, 2012); Non-intentionalism (Shargel, 2015; Whiting, 2011); Representationalism about affective experiences (Mendelovici, 2014; Tye, 2008); Representationalist enactivism (Hufendiek, 2018)	Non-representational enactivism (Hutto, 2012; Shargel & Prinz, 2018)
…Consciously?	(Quasi-)judgmentalism (Nussbaum, 2001; Roberts, 2003; Solomon, 1993); (Quasi-)perceptualism (de Sousa, 1987; Döring, 2007; Goldie, 2002; Helm, 2009; Ratcliffe, 2005; Roberts, 2003; Tappolet, 2016); Representationalism about affective experiences (Mendelovici, 2014; Tye, 2008)	Motivationalism (Scarantino, 2014)	Non-intentionalism (Shargel, 2015; Whiting, 2011); Non-representational enactivism (Hutto, 2012; Shargel & Prinz, 2018); Attitudinalism (Deonna & Teroni, 2012)

Most theorists focus on whether emotions consciously represent evaluative properties. Here, we find a positive answer among (quasi-)judgmentalists and (quasi-)perceptualists and a negative one among enactivists, non-intentionalists, and attitudinalists. To the best of my knowledge, only four of the authors from Table 1 discuss explicitly or implicitly whether emotions represent evaluative properties unconsciously. Among those, Scarantino (2014) and Prinz (2004) endorse the view that they do, while Hutto (2012) and Shargel and Prinz (2018) that they don’t. Only one of these theories—motivationalism—does not seem to specify whether emotions represent evaluative properties consciously.

What I find especially notable about Table 1 is the following: When we ask the unqualified question ‘Do emotions represent evaluative properties?’, we find a strong opposition between the yays and the nays. However, once we take into account consciousness as a variable, the debate is much less polarized. Some hidden compatibilities are revealed: we see that motivationalism is in contradiction neither with (charitable interpretations of) attitudinalism nor non-intentionalism on this question. Indeed, it may well be the case that emotions represent evaluative properties unconsciously but not consciously, thus respecting the claims made by these three theories on the present issue. This also suggests that (charitable interpretations of) attitudinalism and non-intentionalism are compatible with my conclusion although they deny that emotions represent evaluative properties (consciously).^10^ 

### The Relation Between Unconscious and Conscious Representation

The preceding discussion leads to asking: how does the claim that normally elicited emotions represent evaluative properties unconsciously bear on whether they do so consciously?

There certainly is no short answer. This question opens up many contentious issues that require to be dealt with cautiously and this cannot be done in the remainder. I believe nevertheless that the hypothesis that normally elicited emotions represent evaluative properties unconsciously can be a fruitful starting point to discuss whether they do so consciously as well. This starting point, as far as I know, has not been exploited in philosophy. Hopefully, this paper can thus serve as a stepping stone in this direction. In the following, I will only be able to give a foretaste of this task.

Here is one thread that could be followed. Even though appraisals are hypothesized to be unconscious by default, they are said to partly ‘emerge in’ consciousness (see notably Grandjean et al., 2008). For instance, appraisals of low and high coping potentials may ‘emerge’ respectively as conscious feelings of powerlessness (e.g. in fear) and of empowerment (e.g. in pride). Appraisals of goal-unconduciveness and goal-conduciveness may ‘emerge’ as feelings with respectively negative and positive valence. Could that mean that unconscious representations of evaluative properties can become conscious representations? The answer depends on what is the relation between unconscious representations and the corresponding ‘emerged’ feelings.

Here are four potential candidates (some are compatible) for this relation. (1) Causation: unconscious appraisals cause specific feelings—and this cause may play a psychological function, perhaps a representational one as in teleosemantics (relatedly, see Prinz, 2004). (2) Identity: the feeling of, say, positive valence *just is* the feeling of the appraisal of goal-conduciveness. This proposal however threatens to be contradictory with our discussion of the ‘theoretical evidence’ in 3.1: affective scientists don’t consider that we have direct conscious access to appraisals. (3) The third option may eschew this difficulty: it is a relation of identity when and only when our attention is bestowed on unconscious appraisals. We usually don’t have direct conscious access to the latter because, during emotional episodes, we usually don’t/can’t focus our attention on appraisals. But when we can, we feel the appraisals. (4) Metacognition: the conscious feelings would be a higher-order mental state directed at the unconscious representations.

The third and fourth proposals seem to come with the advantages and disadvantages of, respectively, the attentional (e.g. Prinz, 2012) and higher-order (e.g. Armstrong, 1981; Carruthers, 2004; Rosenthal, 1991) theories of consciousness. There certainly are more than four candidates: for instance, Teroni (2021), writing on a related topic, discusses a determinate-determinable and an epistemological relation that may constitute further options.

### The Paradox of Unconscious Emotions

I stated in the introduction that this paper does not take a stance on whether unconscious emotions exist. However, my argument and conclusion may have implications for this debate. To see how, let us consider Arnaud’s (2023) paradox of unconscious emotions:

‘1. Emotions can be unconscious.

2. Emotions essentially have a phenomenology [i.e. a particular phenomenal character].

3. Emotional phenomenology entails emotional consciousness’.

This paradox captures three claims that are *prima facie* plausible but that appear to be incompatible. Arnaud proposes to solve it by a subtle interpretation of the third premise. Her view is that emotional phenomenology designates a kind of conscious experience—a value-loaded perception of the world—that does not require the conscious awareness of one’s emotion. For instance, if Sarah is angry at her sister, this entails a kind of phenomenology: she must perceive her angrily. However, she need not be aware of her emotion in the sense that she may not notice her feeling or realize that she is angry. In such cases, one is thus unaware of undergoing an emotion while this emotional episode nevertheless has a phenomenology. Thus, the emotion is unconscious in some sense (respecting premise 1) although it has a phenomenology (respecting premise 2) that entails a form of consciousness (premise 3).

The possibility of such cases is not in tension with anything that I advance here. However, Arnaud makes some claims in her defense of premise 2 that may be in contradiction with the idea that the appraisals underlying emotions can happen entirely unconsciously. Let me thus say something about this point. This should clarify how this paper bears on the unconscious emotions debate.

Arnaud states that, for an emotion to occur, appraisals must be triggered by a conscious representation of the intentional object. She, for instance, writes: ‘Contrary to discriminating yellow without being consciously aware of it, one cannot be scared by a noise without being consciously aware of that noise.’ (Arnaud, 2023).

One way in which such claims could be in contradiction with mine is if I defended, against premise 2, that there can be emotional episodes without phenomenology. However, I did not make this claim. First, I left it open that the appraisal process possesses a phenomenology even when it remains entirely inaccessible to consciousness, so that the subject is unaware of its phenomenal character (in Block’s terminology, it would have P-conscious properties although it remains A-unconscious). Second, I also left it open that cases where subliminal stimuli are unconsciously appraised result in affective episodes that don’t qualify as emotions (but, perhaps, as moods) so that the term ‘emotion’ would be restricted to cases where the appraised object is consciously accessed (see above 3.2 and Bonard, 2022). For these two reasons, my argument is compatible with premise 2, i.e. that emotions essentially have a particular phenomenal character.

There is another way in which Arnaud’s claims could be in contradiction with mine: if she considered that an appraisal process cannot take place unless some intentional object is *consciously* evaluated positively or negatively. There are passages in her article that could be interpreted as such.^11^ However, it seems not to be what she has in mind when she discusses Winkielman and Berridge (2004)’s experiment. You will remember from above that this experiment involves participants who are subliminally presented with pictures of happy or angry faces and are later asked to taste beverages. Arnaud agrees with me that the subliminal pictures are appraised unconsciously. An appraisal process can thus take place without the intentional object being consciously evaluated. However, she adds an interpretation that I don't make: these unconscious appraisals create an emotion only later, once they are attached, due to a mistaken association, to the beverages, which are consciously evaluated through positive or negative feelings. For Arnaud, the emotion only arises when a valenced feeling is attached to an object consciously accessed. As I stressed in the preceding paragraph, I leave it open whether this is a criterion for emotions. But, in any case, Arnaud and I seem to agree that an appraisal process can take place although no intentional object is consciously evaluated or appraised: this is what happens to Winkielman and Berridge’s participants when they are presented with the subliminal pictures *before* they taste the beverages, before any positive or negative conscious feelings arise.

I have argued that typically elicited emotions involve a component—the appraisal process—that represents evaluative properties unconsciously, although these unconscious representations may later become conscious. My argument leaves it open how we should solve the paradox of unconscious emotions. It is, I think, compatible with Arnaud’s solution, although it is not committed to it, as I leave open options that she rejects. In fact, my argument is also compatible with the rejection of each premise of the paradox. Firstly, I left it open whether appraisals that are never accessed consciously and that don’t create emotional consciousness nevertheless possess an emotional phenomenology—a possibility suggested by e.g. Deonna and Teroni (2012, Chapter 3). Thus, I am not committed to premise 3 (‘Emotional phenomenology entails emotional consciousness’). Secondly, I left it open whether we should call ‘emotions’ mental episodes that lack emotional phenomenology but that possess several of the other emotional components discussed in 2, i.e. appraisal process, action tendency, physiological responses, expressive behavior. If one thinks, as e.g. Clore (1994) does, that such episodes should not be called ‘emotions’, one may be tempted to reject premise 1 (‘Emotions can be unconscious’). If one believes instead, as Kihlstrom et al. (2000), Smith and Lane (2016), or Prinz (2005) do, that such episodes should be called ‘(implicit) emotions’, one may be tempted to reject premise 2 (‘Emotions essentially have a phenomenology’). I have not argued for or against these views.

That being said, I think that Arnaud’s (2023) solution is more elegant, as it allows preserving the three premises of the paradox. My argument nevertheless suggests a slightly different way to preserve these premises since I do not consider, unlike Arnaud, that the appraisal process leading to emotions requires a conscious access to an evaluated object (whether the latter is what Arnaud calls the ‘target’ or ‘the cause’ of the emotion). My conclusion thus suggests that emotional consciousness results from a different process than that suggested by Arnaud. However, developing how exactly this process unfolds and how my claims relate to the different views on unconscious emotions must be left for future work.

### Conclusion

In this section, I hope to have indicated how the conclusion of this paper—that normally elicited emotions represent evaluative properties unconsciously—may lead to fresh and fruitful approaches to various issues in the philosophy of emotion. But, most importantly, I hope that this paper has shown that what emotions unconsciously represent is a question that, by itself, deserves our attention.

## Data Availability

The author does not analyze or generate any datasets, because their work proceeds within a theoretical approach.

## Footnotes

1 I will use as an example the appraisals postulated by contemporary appraisal theorists, but the same reasoning applies for the appraisals postulated by other contemporary psychological theories: we don’t have a direct access to them as part of the flow of our consciousness. For a review of what these other appraisals are, see what Moors (2022, Chapters 4–8) calls ‘evaluation’ in the different theories in question.

2 There arguably are different types of joy, but there is no evidence, as far as I know, that there is a special type of joy whose appraisals would be related to particular objects that require conscious processing, like solving a mathematical problem. Types of emotions are not distinguished by their particular objects but rather by cognitive, motivational, physiological, motor, and phenomenal profiles.

3 By the way, note that, to identify the relevant appraisal process, it does not matter what the person is thinking to themselves *while* they feel joy because what matters is the cognitive process that triggers the joy *before* the feeling of joy emerges—the appraisal process is supposed to give a causal explanation for why a certain type of emotion is elicited. It must thus be an *antecedent* cause to any emotional feeling.

4 If the theoretical and empirical evidence discussed in the preceding paragraphs was misleading and that there were higher-level, conscious, appraisals without unconscious appraisals, then I would need to partially modify my conclusion as follows: emotions represent evaluative properties unconsciously or, in cases with higher-level appraisals without lower-level appraisals, consciously. However, for the reasons discussed, I don’t find this possibility plausible and consider that whenever there are conscious appraisals, there are unconscious appraisals subtending them that represent evaluative properties.

5 These points allow responding to the three challenges to appraisal theory put forward by Schroeter et al. (2015, pp. 370–371), but I will not elaborate on this for reasons of space.

6 If there nevertheless are cases of representation-free emotions, they would constitute an exception to my conclusion. As such, they would join cases where emotions are elicited artificially: emotions where no appraisals take place because they are induced by manipulating the brain through chemical or electrical stimulations or through certain bodily manipulations (for a review, see Moors, 2022, pp. 262–264). However, such hypothetical exceptions, if they exist at all, would seem to be extremely unusual and so I will not count them as normally elicited emotions.

7 This substantial agreement is not universal. E.g. Barrett (2006), a psychological constructivist, considers that emotions are only labelized subjective feelings. There also are appraisal theorists who conceive of appraisals as antecedent causes, but these constitute a small minority (Moors 2022, p. 168). Most appraisal theorists and basic emotion theorists consider appraisals as emotional components (e.g. Ekman and Matsumoto 2009; Ledoux 1989, Panksepp 1998: ch. 3, see also the review by Moors 2022, p. 114–119 and p. 168). And when psychological constructivists focus on appraisals in the present sense, they standardly consider them as emotional components (see e.g. Russell 2003, p. 150, under ‘antecedent event’ and ‘affective quality’) and Ortony and Clore (2015)).

8 ‘Charitable’ in the sense that we could instead take their response to the unqualified question to imply that emotions neither represent evaluative properties consciously nor unconsciously—just like if I say that there are no beers in the fridge, I seem committed to there being neither Belgian nor non-Belgian beers in the fridge.

9 I did not include the content-priority view in Table 1 (e.g. Müller, 2019) because it can be neutral for both questions. Its main claim is that emotions are responses to a prior awareness of evaluative properties: this awareness may or may not be considered by its defenders to be part of what emotions represent (for a discussion, see Mitchell, 2019).

10 In a similar spirit, I present other reasons to consider apparently contradictory theories of emotion to be compatible in Bonard (2021).

11 For instance: “How could I be angry at my sister without being consciously aware of anything about her? In the absence of a conscious representation of something about my sister, no evaluation or appraisal is present, and they are necessary for emotions to occur.” (Arnaud, 2023).
